# New Methods for Processing and Quantifying VO_2_ Kinetics to Steady State: VO_2_ Onset Kinetics

**DOI:** 10.3389/fphys.2017.00740

**Published:** 2017-09-26

**Authors:** Craig R. McNulty, Robert A. Robergs

**Affiliations:** School of Exercise and Nutrition Sciences, Queensland University of Technology, Kelvin Grove, QLD, Australia

**Keywords:** oxygen uptake, mono-exponential, tau, linear regression

## Abstract

Current methods of oxygen uptake (VO_2_) kinetics data handling may be too simplistic for the complex physiology involved in the underlying physiological processes. Therefore, the aim of this study was to quantify the VO_2_ kinetics to steady state across the full range of sub-ventilatory threshold work rates, with a particular focus on the VO_2_ onset kinetics. Ten healthy, moderately trained males participated in five bouts of cycling. Each bout involved 10 min at a percentage of the subject's ventilation threshold (30, 45, 60, 75, 90%) from unloaded cycling. The VO_2_ kinetics was quantified using the conventional mono-exponential time constant (tau, τ), as well as the new methods for VO_2_ onset kinetics. Compared to linear modeling, non-linear modeling caused a deterioration of goodness of fit (main effect, *p* < 0.001) across all exercise intensities. Remainder kinetics were also improved using a modified application of the mono-exponential model (main effect, *p* < 0.001). Interestingly, the slope from the linear regression of the onset kinetics data is similar across all subjects and absolute exercise intensities, and thereby independent of subject fitness and τ. This could indicate that there are no functional limitations between subjects during this onset phase, with limitations occurring for the latter transition to steady state. Finally, the continuing use of mono-exponential modeling could mask important underlying physiology of more instantaneous VO_2_ responses to steady state. Consequently, further research should be conducted on this new approach to VO_2_ onset kinetics.

## Introduction

**Figure 1:**
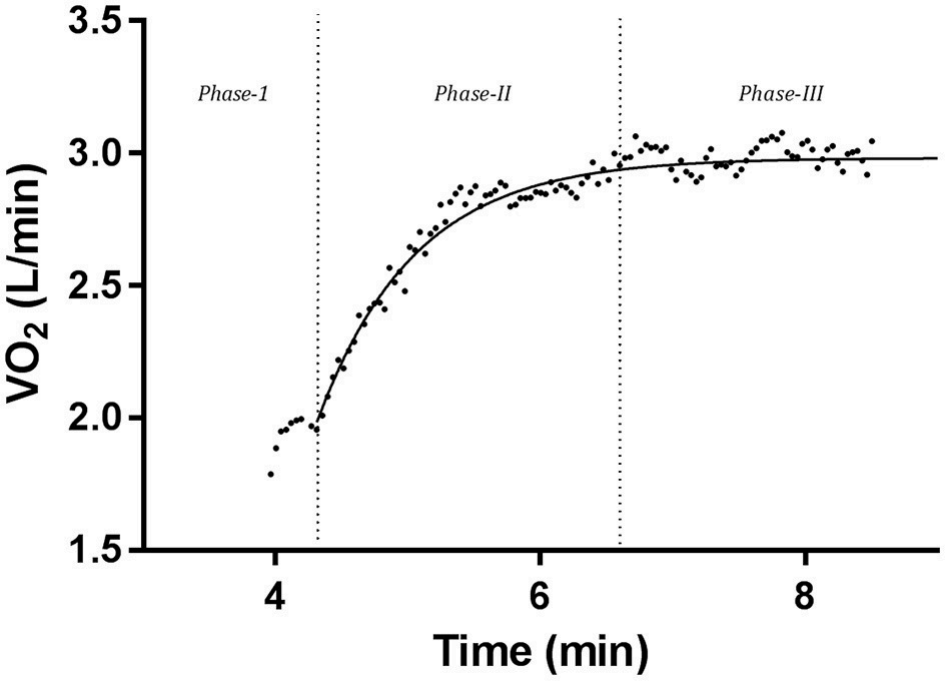
Individual $\dot{\text{V}}$O_2_ kinetics data displaying the three-phase approach to steady state $\dot{\text{V}}$O_2_ following the onset of, or increase in, work load. This bout involved baseline unloaded cycling, followed by a workload increase of 75% VT. Data was averaged at a 7-breath average.

Earlier work in oxygen uptake (VO_2_) kinetics suggested a three-phase approach, from the onset of—or increase in—work to the attainment of steady state VO_2_ in sub-threshold intensities (Whipp et al., 1982) (Figure 1). Phase-I is said to represent the oxygen (O_2_) exchange associated with the initial elevation of cardiac output, and therefore pulmonary blood flow (Jones and Poole, 2005). Phase-II is traditionally identified as an increase toward a steady state VO_2_ following the cessation of phase-I, to be reflective of the increased VO_2_ of contracting muscle and is primarily modeled using a mono-exponential equation (Whipp and Ward, 1990). Finally, phase-III indicates the attainment of steady state VO_2_ (Whipp et al., 1982).

As stated, phase-II is modeled using a mono-exponential equation (Equation 1) incorporating a time constant (tau; τ) that was originally adapted to VO_2_ kinetics data processing by Whipp (1971). This method has been used almost exclusively to process VO_2_ kinetics data over the past 45 years (Linnarsson, 1974; Hughson and Morrissey, 1982; Barstow et al., 1993; Brittain et al., 2001; Spencer et al., 2013). The equation, which is sometimes slightly modified to suit study methodologies (for example, the inclusion or exclusion of a time delay), is generally presented as:

(1) $$\Delta \text{Y}(t)=\Delta \text{Y}(\text{ss})\{1-e^{-1(t-\text{TD})/\tau }\}$$

where Y represents VO_2_; ΔY(*t*) is the increase in Y above the prior steady state value at time *t*; ΔY(ss) is the steady state increase in Y; TD is the phase-I time delay; and τ is the time constant of the response.

This method of data processing, although extensively used in research, has at times displayed inconsistencies in past research. In particular, phase-II kinetics are ascribed to a first order mechanism (i.e., follow a time constant that is invariant across increasing exercise intensities). Some earlier research has discussed irregularities with this interpretation (Linnarsson, 1974; Casaburi et al., 1989), while Hughson and Morrissey (1982) and Koppo et al. (2004) identified a clear slowing (increasing τ) of phase-II kinetics to steady state. They claimed that a three-phase model of VO_2_ kinetics was only useful when all three phases are very clearly defined. This is often not the case, and is highly dependent on subject's health status, fitness, and comfort with the study's mode of exercise (Weltman and Katch, 1976; Spiro, 1977; Hickson et al., 1978; Nery et al., 1982). Stirling et al. (2005) also stated that it was unclear whether the three distinct phases would provide a reasonable model of the underlying physiological processes.

**Figure 2:**
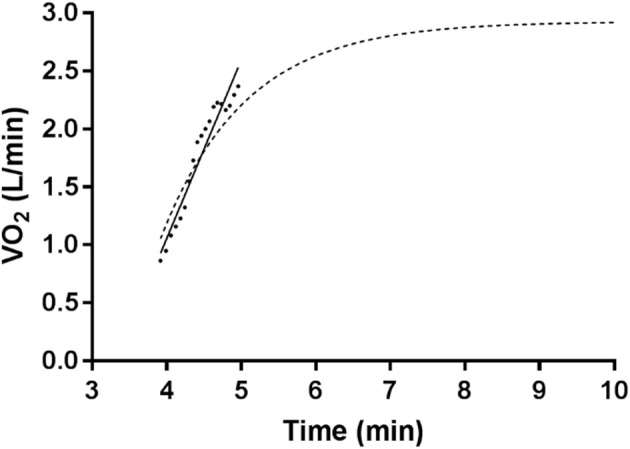
Single bout $\dot{\text{V}}$O_2_ data with a 90% VT increase from unloaded cycling, fitted with a linear regression to the onset segment, and overlayed with a mono-exponential model (dashed line) without a time delay from the parameters of the entire data set.

Through work in the field, the authors have identified a trend within the VO_2_ data which they believe needs to be further assessed. From the termination of phase-I (where it exists in the data), until the beginning of a non-linear response in phase-II, there is a clear linear pattern within the data. The authors have termed this initial linearity as “onset kinetics”. As can be seen in Figure 2, the onset kinetics follow a previously non-modeled linear approach for the first ~1 min following the initiation of phase-II.

Given that past researchers have acknowledged that the VO_2_ kinetic response to increases in steady state workload is quite complex (Hughson and Morrissey, 1982; Koppo et al., 2004; Stirling et al., 2005; Robergs, 2014; McNulty et al., 2015), and that the initial onset response (see Figure 2) appears to be linear, there is merit to investigate the linear onset kinetics of VO_2_ during exercise transitions to steady state. Consequently, the aims of this paper were to (a) compare a linear regression fit of the onset kinetics to a mono-exponential fit of the data, (b) compare the mono-exponential model fit for phase-II to a linear regression fit for the onset kinetics + mono-exponential fit to the remainder of phase-II.

## Materials and methods

### Participants

Ten male subjects (mean age = 28 ± 9 years; height = 178.4 ± 7.7 cm; weight = 76.9 ± 11.3 kg) were recruited and completed the exercise trials of this study. The criteria for recruitment were healthy males aged between 18 and 45 years, and free from musculo-skeletal injury, or the presence of cardio-pulmonary and metabolic disease or more than two risk factors for disease. In addition, each participant was recruited on a basis of self-reported physical fitness (the minimum requirements for recruitment purposes were current endurance training for at least 45 min, three times per week), with a measured VO_2max_ ≥ 40 mL/kg/min. Recruitment occurred at a country NSW university, as well as local gymnasiums. All participants were asked to complete an Exercise and Sports Science Australia: Adult Pre-Screening System tool (Exercise and Sports Science Australia, 2014) to determine that they were in good physical health with no musculoskeletal disorders or risk factors for sedentary lifestyle diseases. Written informed consent was obtained from each participant prior to data collection and all methods were approved by the institution's Human Research Ethics Committee.

### Familiarization and baseline testing

After completion of informed consent, a familiarization session, as well as a VO_2_ maximum ramp protocol cycle ergometer test, were administered for each participant. During the familiarization session, the subject's height and mass were recorded, and the cycle ergometer's seating and handle bar arrangement were adjusted for each subject's preference and biomechanical needs. These adjustments were recorded and maintained for all future bouts. Before exercising, the subjects were asked to remain seated for 5 min in order to ascertain a resting HR measure. The subjects were then asked to cycle at 100 W for several minutes until they had established a comfortable, and constant pedaling cadence. This cadence was the set point for the entirety of the testing for that individual subject.

Prior to conducting the VO_2_ ramp test (and for all subsequent trials) the subject was fitted with a multiple one-way valve mouthpiece system supported by an acrylic head unit. Electrocardiography (ECG) was used to acquire heart rate throughout the VO_2_ max test and trials using a 5-lead ECG configuration (CASE, GE Healthcare, Waukesha, USA). The ECG leads were attached using gel electrodes placed over the spine of both scapulae, the iliac crest of both ilia, and between the 4th and 5th intercostal space along the mid-axillary line of the left side of the torso. For indirect calorimetry, expired gas analysis was acquired using a 3 L latex compliant and elastic mixing bag placed on the expired port of the mouthpiece, and mixed expired air was sampled continuously and pumped to rapid response O_2_ and carbon dioxide (CO_2_) gas analysers (AEI Technologies, Pittsburgh, PA, USA). During and following each breath, the elastic recoil of the mixing bag caused air to be vented through a 1 cm diameter hole in the inferior end of the mixing bag. Expired gas signals were acquired for 100 ms at the start of each inspired breath and aligned to the timing of end expiration based on a pre-determined measured time-delay. Ventilation was measured by a flow turbine (UVM, VacuMed, Ventura, CA, USA) connected to the inspired side of the mouthpiece. All data were acquired using custom developed software (LabVIEW™, National Instruments, Austin, TX) and commercial electronic acquisition devices (National Instruments, Austin, TX). The breath-by-breath system was calibrated before the ramp test and before each bout in both trials using a 3 L syringe and commercial medical grade calibration gas (16.00% O_2_ and 5.00% CO_2_). These methods have been validated and described in more detail elsewhere (Kim and Robergs, 2012).

Administration of the VO_2_ ramp test had the subject cycle at their predetermined cycling cadence, for which they were asked to maintain for the entire test. The ramp function for each subject was based on their self-reported endurance fitness, and the need to constrain the test to between 8 and 12 min (Buchfuhrer et al., 1983; Astorino et al., 2005; Yoon et al., 2007), and consequently varied between 25 and 35 W/min between subjects. The VO_2_ ramp protocol consisted of 2 min of rested breathing (to attain a baseline reading), followed by 2 min at double the ramp function Watts, and then followed by a near continuous ramp function (increment at 0.5 Hz). The subjects were also instructed to continue cycling until volitional exhaustion which was defined by a decrease in cadence <40 rev/min (Astorino et al., 2000).

Using the breath-by-breath VO_2_ data collected from the ramp test, the VT of each subject was determined visually by the ventilatory equivalent method (Gaskill et al., 2001) using a custom designed computer program (LabVIEW™, National Instruments, Austin, TX, USA). The VT was detected by the program through the user directed application of three linear segments to the data. The VT was computed as the time of the intersection between segment 1 (baseline response, slope ~ 0) and segment 2 (initial deviation from baseline). The detection of the VT required agreement between two investigators (agreement was set at ± 10 s). Where there was opposing detection, a third researcher was asked to interpret the data. The VT was then used to determine to cycle ergometer power output required for the two exercise trials.

### Exercise protocol

Research on phase-II VO_2_ kinetics has only used a minimal number of magnitude increases (Whipp, 1971; Whipp et al., 1982; Whipp and Ward, 1990; Barstow et al., 1996). Therefore, a multiple-bout cycling trial was developed. The trial involved five separate bouts of unloaded cycling to different increments.

Following a 5 min warm-up at 50 W on the cycle ergometer, each participant was again fitted for indirect calorimetry and ECG. The cycling trial involved five bouts to differing magnitudes from unloaded cycling, with 15 min of seated rest between each bout to limit trial-to-trial variability due to post-exercise O_2_ consumption (Borsheim and Bahr, 2003). The magnitudes in order of intensity were: 30, 45, 60, 75, and 90% of VT. The trial required the subject cycle at an unloaded magnitude for 2 min before completing 10 min at the increased magnitude. The participants were instructed to maintain the same comfortable cycling cadence for each bout despite the electronic ergometer adjusting resistance with changed cadence to ensure a stable power output. The order of administration of each magnitude were determined by a Latin Squares design (Kirk, 2010). A minimum time-frame of 48 h separated the completion of the VO_2_ ramp test and the multiple-bout exercise trial.

### Data reduction and analysis

The raw breath-by-breath data, which included absolute and relative VO_2_, respiratory exchange ratio, and the ventilatory equivalent ratios for O and CO_2_, were processed using a 7-breath average from custom designed software (LabVIEW™, National Instruments, Austin, TX). Each trial text file was imported into a commercial graphics and curve fitting program (Prism, GraphPad Software, La Jolla, CA, USA), and data were removed for the initial rest data collection of each trial. Data were then graphed and the phase-I VO_2_ data were identified and then also removed for each trial. Initially, the whole data sets (phase-II and -III) were fitted using the mono-exponential function of Equation (1).

The onset VO_2_ kinetics for each data set were fitted with a linear regression. The onset kinetics were visually identified separately by both authors. The onset data were defined as the data points which follow a near-linear fashion from the onset of phase-II (or the onset of exercise if there was no clear phase-I), until an evident decline in linearity within phase-II. The remainder data, in a separate Prism file, were then fitted with Equation (1).

The onset data were then modeled with Equation (1), using the parameters set by the application of the model to the entire data set (phase-II and -III). From here, standard error of the estimate (S_y.x_) and coefficient of determination (*R*^2^), as well as their respective confidence intervals, between both linear and mono-exponential modeling were recorded.

The remainder segment of data were fit with a new application of Equation (1) and the model of the entire data set, again using the set parameters. S_y.x_ and *R*^2^, as well as their respective confidence intervals, between both non-linear data fits were recorded.

### Statistical analyses

Statistical analysis of the data was performed using SPSS 23 (IBM Corporation, New York, NY, USA). The subjects of this study completed five cycling bouts of varying intensity (30, 45, 60, 75, and 90% VT). The data was processed in order to ascertain S_y.x_ and *R*^2^ for VO_2_.

To assess the goodness of fit between a linear regression vs. mono-exponential (non-linear) modeling approach to the onset VO_2_ data, two separate analysis of variance (ANOVA) were used. A two-way ANOVA (INTENSITY [5] × METHOD [2]) was implemented to analyse the S_y.x_ for both linear, and non-linear models. Following this, a second two-way ANOVA (INTENSITY [5] × METHODS [2]) was used to analyse the *R*^2^ values of the data for both linear and non-linear models.

Following the identification and analysis of the onset VO_2_ kinetics data, there remains the latter-end of the phase-II breath-by-breath data to phase-III, which continues up until the termination of each exercise bout. This data has been labeled “remainder” by the authors. Another set of ANOVAs were used to analyse both the S_y.x_ and *R*^2^ of the remainder data to assess goodness of fit of two non-linear models. A two-way ANOVA (INTENSITY [4] × METHOD [2]) was implemented to analyse the S_y.x_ for both a non-linear model applied to the remainder data, and the full mono-exponential model (from the entire data set) fitted to the remainder data. Following this, a second two-way ANOVA (INTENSITY [4] × METHODS [2]) was used to analyse the *R*^2^-values of the data for both of these fits. Note the 30% intensity data was omitted from the analysis of the remainder as the relatively low increase in power output (and therefore ΔVO_2_) would not allow for mono-exponential modeling of most subject data.

Finally, each individual power output (30, 45, 60, 75, and 90% VT) of all bouts for each subject—presented as an absolute Watts value—was graphed against the respective slope (L/min^−2^) of the VO_2_ onset kinetics.

Significance was set at *p* < 0.05. All data are presented as mean ± SD.

## Results

**Figure 3:**
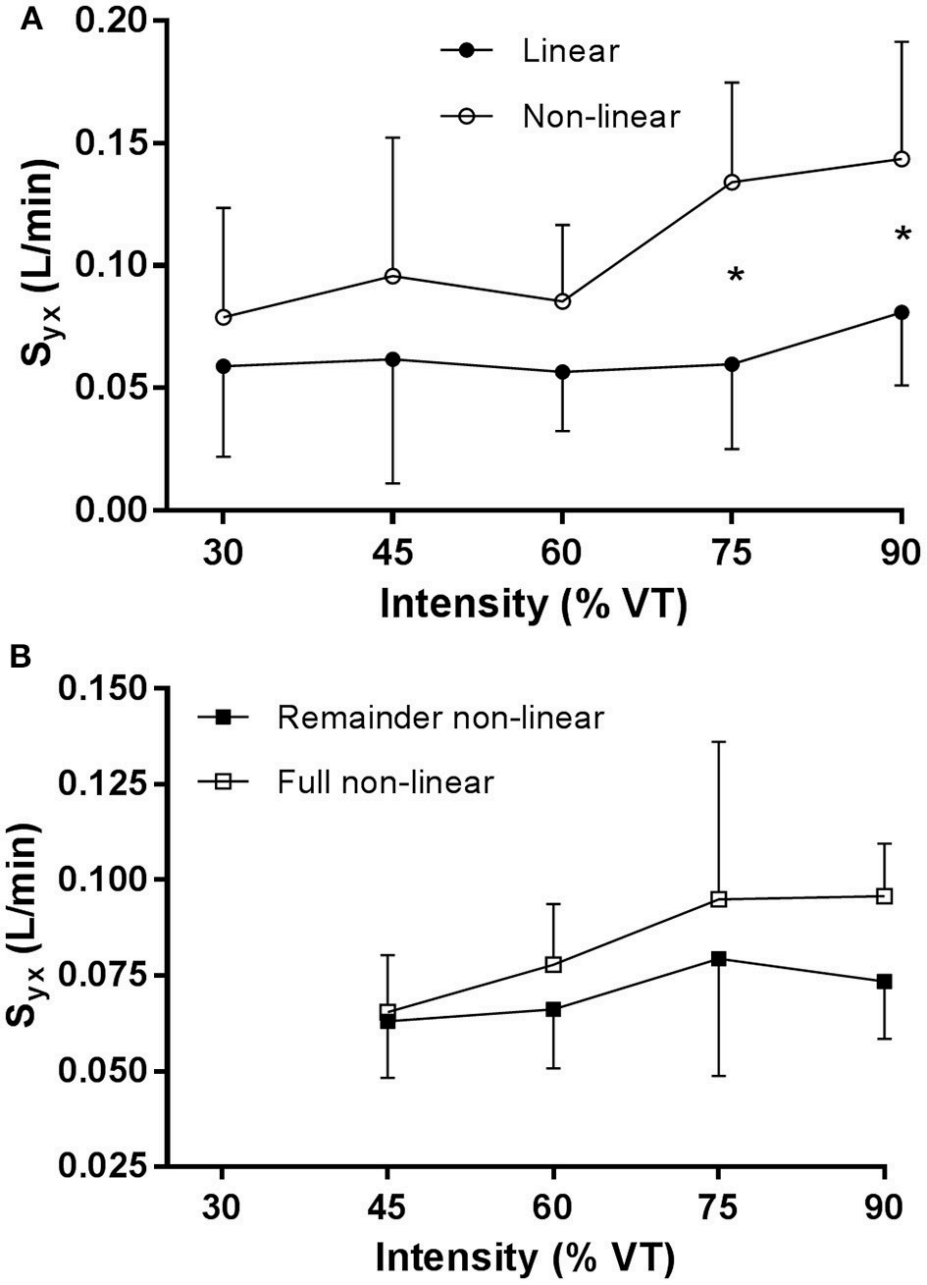
**(A)** S_y.x_ $\dot{\text{V}}$O_2_ data for the linear regression model and non-linear model for the onset kinetics data, and **(B)** S_y.x_ $\dot{\text{V}}$O_2_ data for the remainder data and the full mono-exponential fitted to the remainder data. ^*^p < 0.05.

For the mean onset linear and non-linear VO_2_ data in Figure 3A, S_y.x_ was significantly lower (main effect, *p* < 0.001) with the linear model fit compared to the non-linear fit. There was a significant (*p* = 0.02) main effect for exercise intensity (30, 45, 60, 75, and 90% VT) and a significant interaction (*p* = 0.04) between exercise intensity and method, where the increase in S_y.x_ for the non-linear model fit was significant for exercise intensities above 60% VT.

For the mean remainder, non-linear and full mono-exponential VO_2_ data in Figure 3B, S_y.x_ was significantly lower (main effect, *p* < 0.001) with the non-linear model fit compared to the full mono-exponential fit. There was no significant main effect for exercise intensity. There was no significant interaction between exercise intensity and method.

**Figure 4:**
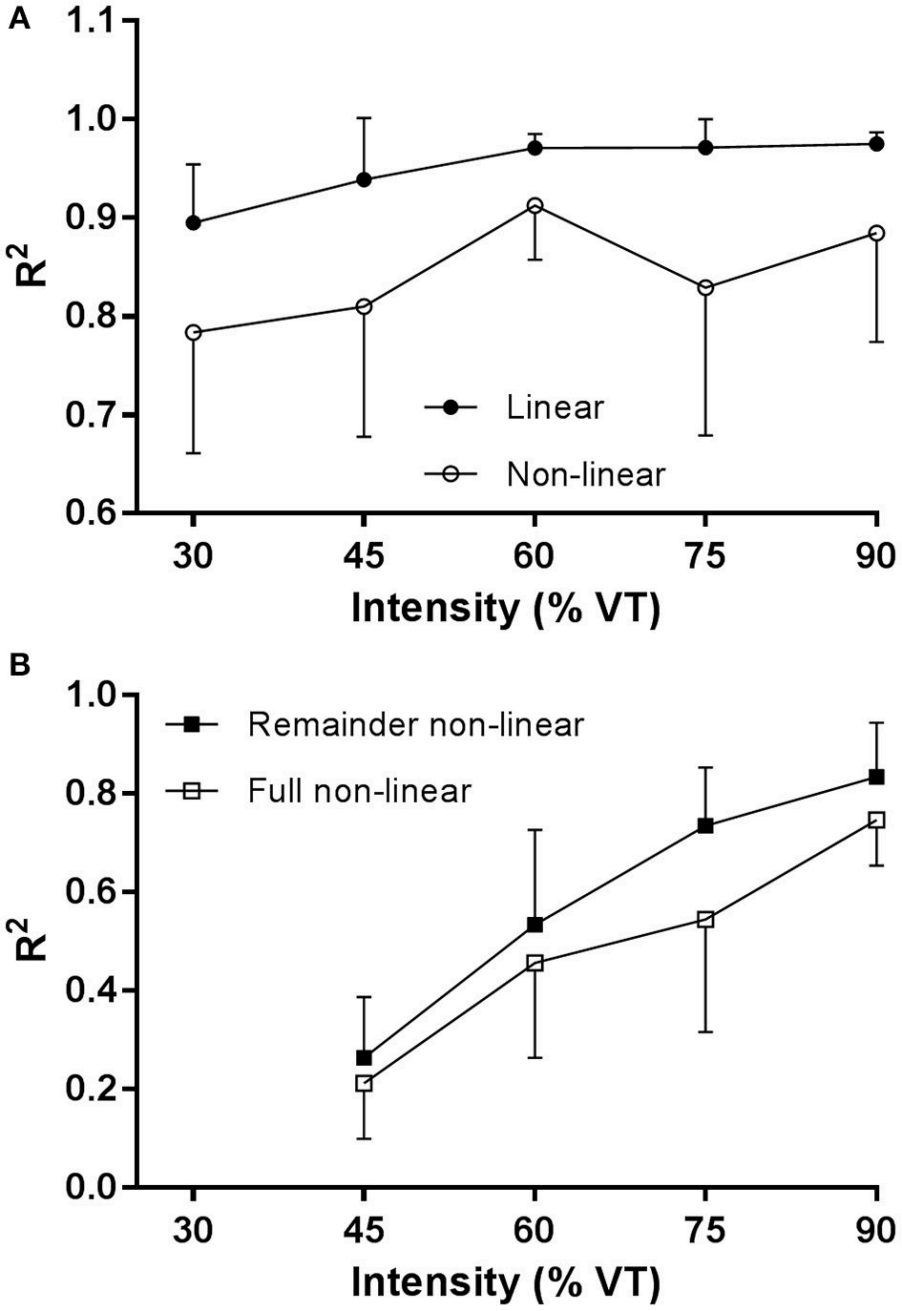
**(A)** *R*^2^ $\dot{\text{V}}$O_2_ data for the linear regression model and non-linear model for the onset kinetics data, and **(B)** *R*^2^ $\dot{\text{V}}$O_2_ data for the remainder data and the full mono-exponential fit to the remainder data.

For the mean onset linear and non-linear VO_2_ data in Figure 4A, *R*^2^ was significantly (main effect, *p* < 0.001) higher with the linear fit compared to the non-linear fit. There was a significant main effect (*p* = 0.02) for exercise intensity (30, 45, 60, 75, 90% VT). There was no significant interaction between exercise intensity and method.

For the remainder non-linear and full mono-exponential VO_2_ data in Figure 4B, *R*^2^ was significantly higher (main effect, *p* < 0.001) with the non-linear fit compared to the full mono-exponential fit. There was a significant (main effect, *p* < 0.001) effect for exercise intensity (45, 60, 75, 90% VT). There was no significant interaction between exercise intensity and method.

**Figure 5:**
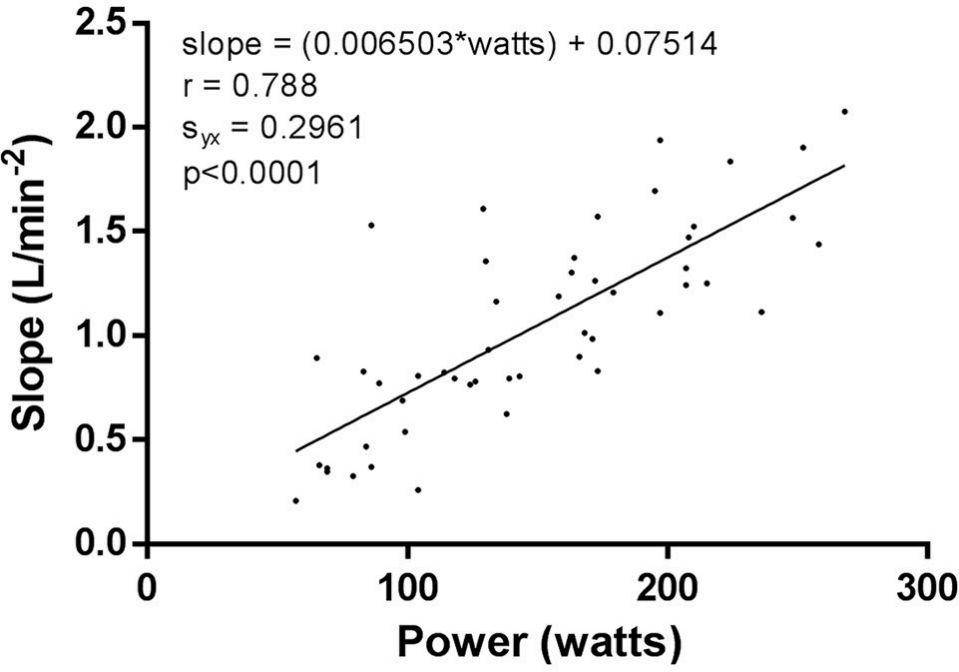
Linear regression of the linear onset data for power vs. slope of all subjects at all exercise intensities.

Finally, Figure 5 presents the power (Watts) vs. slope (L/min^−2^) data for all subjects and all exercise bouts (30, 45, 60, 75, and 90% VT) for the onset linear regression fit. The regression data are incredibly invariant, revealing that despite subjects of differing fitness, the variability in linear onset kinetics was 62% explained by absolute power output (Watts) alone.

## Discussion

The primary aim of this study was to compare modeling techniques for VO_2_ kinetics. Therefore, we compared linear and non-linear modeling of the onset VO_2_ kinetics of the phase-II approach to steady state for multiple exercise transitions. We also compared the fit of the remainder VO_2_ kinetics using two different mono-exponential models. In doing so, we were able to statistically ascertain the appropriateness of using traditional non-linear modeling techniques of phase-II VO_2_ kinetics to steady state vs. a new method based on the linear fit of the initial ~30 s of phase-II data for these exercise transitions.

The main findings of our results are that there was a significantly decreased error (S_y.x_) and significantly increased goodness of fit for the linear regression applied to the onset VO_2_ kinetics, when compared to the traditional non-linear fit. This pattern was also evident in the remainder segment of VO_2_ data. Lastly, slope data (L/min^−2^) for each onset VO_2_ kinetics linear regression fit was plotted against the respective cycling power (Watts) for that particular exercise transition. This was completed for each bout (30, 45, 60, 75, and 90% VT) for each participant. This data was then fitted with a linear regression. Results displayed a strong correlation between absolute power output and the speed of onset kinetics.

Currently, the majority of combined phase-II and phase-III VO_2_ data are fitted using a single mono-exponential function to derive a time constant (tau, τ) to quantify the rate of the non-linear kinetics (Whipp, 1971; Linnarsson, 1974; Hughson and Morrissey, 1982; Barstow et al., 1993; Brittain et al., 2001; Spencer et al., 2013). In this context, τ represents the time to attain 63.2% of the VO_2_ increment for the exercise transition. Whipp (1971) first introduced the use of the mono-exponential equation in an attempt to quantify phase-II VO_2_ kinetics. Whipp soon became the leading authority on VO_2_ kinetics research, publishing numerous papers (which adopted the mono-exponential method) alongside his doctoral mentor and a number of other colleagues (Whipp, 1971, 1987; Whipp and Wasserman, 1972; Whipp et al., 1982; Whipp and Ward, 1990). The use of a mono-exponential model to fit VO_2_ kinetics data has since been used widely, and almost exclusively, in VO_2_ kinetics to steady state (and non-steady state) research.

As previously explained, we are concerned that there has been minimal constructive criticism of single mono-exponential modeling of VO_2_ data during exercise transitions to steady state. Despite more than 40 years since the original proposed research for adopting a single mono-exponential modeling of phase-II VO_2_ kinetics, we have only been able to find a limited number of studies that have provided empirical evidence expressing disagreement with such practice (Hughson and Morrissey, 1982; Brittain et al., 2001; Koppo et al., 2004; MacPhee et al., 2005; Stirling et al., 2005; McNulty et al., 2015) Hughson and Morrissey (1982) showed a slower VO_2_ kinetic response with a work-to-work exercise transition, compared with a rest-to-work transition. Research following this discussed a slower kinetic response (and therefore a slowed τ) with increasing exercise increments within the moderate intensity domain (Brittain et al., 2001; Koppo et al., 2004; MacPhee et al., 2005). McNulty et al. (2015) further confirmed these findings with their assessment of the VO_2_ kinetic response across a broad range of sub-threshold exercise intensity transitions. Finally, Stirling et al. (2005) assessed the mono-exponential equation (amongst other concepts) on a very mathematical platform. They argued that a mono-exponential function, as part of a 3-phase approach to VO_2_ kinetics data to steady state, is a vast oversimplification of the physiological responses. This is also in support of conclusions of the inferiority of the mono-exponential model. Following on from this, Stirling et al. (2005) suggested that modeling the data with a continuous differential function would be more suitable.

### VO_2_ onset segment kinetics

Traditionally, the phase-II VO_2_ kinetic response has been modeled using a mono-exponential function (Whipp, 1971). Unfortunately, this model was never expressed as a theory nor exposed to empirical validation or falsification, yet for the last 45 years it has been accepted as the sole method for use in quantifying the kinetics of the change in VO_2_ during exercise transitions to steady state. Contrary to convention, our results indicated that a linear regression is a more appropriate method of modeling onset VO_2_ kinetics data.

Treating kinetics as a linear function has extensive application in different disciplines of physiology. The contractile rate of force development describes the ability and rate of a muscle to develop muscular force. This is measured as the linear slope of the force-time curve (Andersen and Aagaard, 2006; Li et al., 2015) and is focused primarily on the very early onset of muscular contraction. From a mathematical viewpoint, time envelopes are used to ascertain linear fits of the muscle force changes over time to quantify the rate of muscle force development (Andersen and Aagaard, 2006; Robergs, 2014). We believe that there is important knowledge of systemic and muscle exercise physiology to be gained from quantifying these more instantaneous kinetics separate to a mono-exponential function that is dependent on the total data set, and hence we have termed this initial linear phase of the exercise transition response as onset kinetics. Such an approach not only has relevance to VO_2_, but also other measures of human physiology such as heart rate, ventilation, tidal volume, breathing frequency, muscle oxygenation, central, and peripheral blood flow, etc.

The added relevance of the onset kinetics segment is seen in Figure 5 where we compared the linear onset slope data (L/min^−2^) to the respective absolute power output (Watts) of each bout. Our results indicated a significant increase in slope as absolute exercise intensity increased, and more importantly, a remarkably consistent response across all subjects and exercise intensities. This concept has been previously introduced by Robergs (2014) through a computer simulation. A linear regression was fitted to the initial 30 s of the phase-II response for six exercise transitions (VO_2_ plateaus of 0.5, 1.0, 1.5, 2.0, 2.5, and 3.0 L/min). The resulting slope data for each respective exercise transition became progressively steeper at 0.18, 0.54, 0.90, 1.25, 1.61, and 1.97 L/min^−2^). The onset slope data findings in this study further support this prior work, and the uniformity of this response might reveal that the onset kinetics response is largely driven by the motor unit recruitment of the increment in exercise intensity of the exercise transition, with minimal dependence on other subject features such as age, sex, endurance fitness, etc. Such evidence reveals the possible enhanced physiological importance of the subsequent (remainder) non-linear response to steady state. The content we have discussed here reveals directions and topics for future research in VO_2_ kinetics modeling.

### VO_2_ remainder segment kinetics

If the onset kinetics is surprisingly less variable between subjects, then the greatest contribution to determining the between subjects variability of the mono-exponential model might be in the determinants to the remainder segment of the response. It is logical to conclude that the onset kinetics is primarily governed by motor unit recruitment factors. The added features of determinants to pulmonary VO_2_ kinetics as defined by Robergs (2014) may be revealed in this slower non-linear response. Such factors include: cardiac output, muscle blood flow, muscle fiber type, muscle VO_2_ kinetics, and ventilation. Further investigation into the remainder kinetics (as well as the linear onset kinetics) in different and special populations, could have added benefit to understanding the VO_2_ kinetic response.

### A new theory of VO_2_ kinetics during exercise transitions to steady state

The debate surrounding the physiological basis of VO_2_ on-kinetics to steady state is ongoing. There has been much experimentation and commentary discussing O_2_ delivery and utilization (Hughson and Morrissey, 1982; MacDonald et al., 1997; Grassi et al., 1998; Burnley et al., 2000; Koppo et al., 2004), motor unit recruitment (Cleuziou et al., 2004), muscle fiber type (Barstow et al., 2000), muscle phosphocreatine (Binzoni et al., 1992; Barstow, 1994; Rossiter et al., 2002), and oxidative phosphorylation (Korzeniewski and Zoladz, 2006) in an attempt to formulate a conclusive theoretical framework for explaining VO_2_ kinetics to steady state. During this time, a mono-exponential function has been widely accepted to model the phase-II VO_2_ kinetics as a first-order linear system (Whipp, 1971). This has also come under enquiry, as its basis of predicting phase-II kinetic behavior has been shown to be inaccurate in some experimentation (Hughson and Morrissey, 1982; Brittain et al., 2001; Koppo et al., 2004; McNulty et al., 2015). As well, the use of a potentially over-simplified model as such combines numerous contributing responses into a single parameter estimate which is likely not attributed to distinct physiological systems (Bakker et al., 1980). Despite this, the mono-exponential equation (and τ) has been continually applied to the majority of VO_2_ kinetics data processing. As well, the mono-exponential application has proceeded into other aspects of kinetics processing in regards to exercise transitions to steady state. Intramuscular phosphocreatine kinetics (Rossiter et al., 1999), de-oxygenated hemoglobin kinetics (DeLeroy et al., 2003; MacPhee et al., 2005), and muscle blood flow kinetics (MacPhee et al., 2005) have all been processed using a mono-exponential formula, again without any critical empirical investigation.

We propose that the VO_2_ response to exercise transitions to steady state is more complex than a mono-exponential function. It is clear that there is still much research to be completed concerning the physiological processes, and appropriate modeling, of the VO_2_ kinetic response to exercise transitions to steady state. The question needs to be raised: how can we blindly follow a mathematical model of such physiological responses, when there is still ongoing debate regarding the underlying physiology itself? As scientists, we need to be open to new fundamental ideas and compelling research as they present themselves.

## Conclusions and recommendations

Historically, VO_2_ kinetics research has been focused almost primarily on complete phase-II non-linear modeling of exercise transition to steady state. This may be viewed as a somewhat over-simplification of the response kinetics, more attuned to fitting the data to a chosen model rather than ascertaining the best model to fit the data. Our results indicated that a linear regression fitted to the initial (first ~30 s of exercise transition) phase-II kinetics is more appropriate than a mono-exponential function. Further investigation into the VO_2_ kinetic response, as well as other similarly modeled responses (phosphocreatine, de-oxygenated hemoglobin, limb blood flow), and in varied populations, may yield a better understanding of the complimentary effects of the numerous physiological responses underpinning the VO_2_ kinetic response to exercise transitions to steady state.

## Ethics statement

This study was carried out in accordance with the recommendations of the Charles Sturt University Human Research Ethics Committee with written informed consent from all subjects. All subjects gave written informed consent in accordance with the Declaration of Helsinki. The protocol was approved by the Human Research Ethics Committee.

## Author contributions

CM and RR designed the study. CM collected data. CM and RR analyzed the data. CM wrote the manuscript. RR edited the manuscript. CM and RR read and approved the final manuscript.

### Conflict of interest statement

The authors declare that the research was conducted in the absence of any commercial or financial relationships that could be construed as a potential conflict of interest.
